# SHIFTING SOCIAL PARTICIPATION AND LEISURE PATTERNS AMONG CHINESE OLDER ADULTS: EVIDENCE FROM NATIONAL SURVEYS

**DOI:** 10.1093/geroni/igae098.1968

**Published:** 2024-12-31

**Authors:** Yu Fang, Zheng Ouyang

**Affiliations:** China Research Center on Aging, Beijing, Beijing, China (People’s Republic); China Research Center on Aging, Beijing, Beijing, China (People’s Republic)

## Abstract

Based on the data of “The National Survey on Older Persons in Urban and Rural China” conducted in 2000, 2006, 2010 and 2015, the paper examines the changing dynamics of social participation and leisure activities among Chinese older adults. Findings from the 2015 survey indicate a notable increase in the participation rate of public welfare activities among the older adults, with nearly half actively involved in various community endeavors. Particularly, urban areas showed enhanced participation, contributing to individual fulfillment and societal harmony. Moreover, a significant proportion of older adults expressed willingness to assist peers in need, underscoring the prevalence of mutual support networks within communities. Additionally, the development of Older Person’s Associations (OPA) has gained momentum, serving as vital platforms for older people self-management and social engagement. However, challenges persist, with disparities in participation rates between urban and rural areas. While static leisure activities remain prevalent, there is a notable rise in Internet usage among the older adults, offering avenues for social interaction and information dissemination. Despite advancements in spiritual and cultural engagement, attention to mental health among the older adults remains crucial, especially in addressing issues like suicide and mental health concerns among the rural older people population. The paper emphasizes the importance of bolstering support systems and services to promote holistic well-being among the aging population.

